# Antileishmanial Lead Structures from Nature: Analysis of Structure-Activity Relationships of a Compound Library Derived from Caffeic Acid Bornyl Ester

**DOI:** 10.3390/molecules19021394

**Published:** 2014-01-27

**Authors:** Jan Glaser, Martina Schultheis, Sudipta Hazra, Banasri Hazra, Heidrun Moll, Uta Schurigt, Ulrike Holzgrabe

**Affiliations:** 1 Institute of Pharmacy and Food Chemistry, University of Würzburg, Am Hubland, Würzburg 97074, Germany; E-Mail: jan.glaser@uni-wuerzburg.de; 2 Institute for Molecular Infection Biology, University of Würzburg, Josef-Schneider-Str. 2, Würzburg 97080, Germany; E-Mails: martina.schultheis@uni-wuerzburg.de (M.S.); heidrun.moll@uni-wuerzburg.de (H.M.); 3 Department of Pharmaceutical Technology, Jadavpur University, Kolkata-700032, India; E-Mails: sudipta_manu@rediffmail.com (S.H.); banasrihazra@yahoo.co.in (B.H.)

**Keywords:** *Valeriana wallichii*, leishmaniasis, caffeic acid bornyl ester, structure-activity relationship

## Abstract

Bioassay-guided fractionation of a chloroform extract of *Valeriana wallichii* (*V*. *wallichii*) rhizomes lead to the isolation and identification of caffeic acid bornyl ester (**1**) as the active component against *Leishmania major* (*L*. *major*) promastigotes (IC_50_ = 48.8 µM). To investigate the structure-activity relationship (SAR), a library of compounds based on **1** was synthesized and tested *in vitro* against *L. major* and *L. donovani* promastigotes, and *L. major* amastigotes. Cytotoxicity was determined using a murine J774.1 cell line and bone marrow derived macrophages (BMDM). Some compounds showed antileishmanial activity in the concentration range of pentamidine and miltefosine which are the standard drugs in use. In the *L. major* amastigote assay compounds **15**, **19** and **20** showed good activity with relatively low cytotoxicity against BMDM, resulting in acceptable selectivity indices. Molecules with adjacent phenolic hydroxyl groups exhibited elevated cytotoxicity against murine cell lines J774.1 and BMDM. The Michael system seems not to be essential for antileishmanial activity. Based on the results compound **27** can be regarded as new lead structure for further structure optimization.

## 1. Introduction

Leishmaniasis is one of the most dreadful infectious diseases worldwide. It is endemic in 98 countries and almost 350 million people on five continents are at risk of infection [1,2]. The disease has an especially high impact on the population of the disadvantaged regions of South America, Africa and Asia (especially India), all of them suffering from poverty and low health care standards. Leishmaniasis is caused by protozoan parasites, which are transmitted by the bite of the sand fly. It comprises a variety of diseases. Cutaneous leishmaniasis mostly caused by *L. major* produces skin sores, ulcers and eventually heals by leaving defacing scars. The most severe form is the visceral leishmaniasis also known as *kala-azar* or black fever. Here the parasites invade internal organs, especially the liver and the spleen. This is fatal if left untreated. The species known to cause visceral leishmaniasis is *L. donovani* which is prevalent in India and East Africa. 70% of the global burden of *kala-azar* is concentrated in India [1,3]. The estimated incidence of visceral leishmaniasis in India is 146,700 to 282,800 cases per year [1].

The infection can be treated but the drugs in use like miltefosine or amphotericin B show severe side effects. Additionally these drugs are expensive and require adequate medicinal care which is not readily available in the most affected regions of India or Africa. Therefore, the need for new antileishmanial drugs is evident. As reported previously the chloroform extract of *V. wallichii* roots showed antileishmanial activity [4]. In this paper we describe the isolation and structure elucidation of one active ingredient, which was assigned to be caffeic acid bornyl ester (**1**). In order to analyse structure-activity relationship for activity optimization a library of derivatives has been synthesized. The respective cytotoxicity against diverse species of *Leishmania* promastigotes, amastigotes, and macrophages was determined.

## 2. Results and Discussion

### 2.1. Isolation

The crude chloroform extract obtained from the pulverized rhizomes of *V. wallichii* was subjected to bioactivity-guided fractionation. The extract was roughly fractionated into twelve fractions by means of semi-preparative HPLC using reverse phase material and a gradient of H_2_O/MeOH as the eluent. The most active fraction was repeatedly partitioned by normal phase silica gel column chromatography with CHCl_3_/MeOH (4.8:0.2 *v/v*) as mobile phase. From a relatively polar sub-fraction with an IC_50_ of 11.8 µg/mL against *L. major* promastigotes a pure compound could be isolated. ^1^H- NMR data showed two doublets for vinyl protons at 6.3 and 7.6 ppm, respectively, with the typical coupling constants of 15.9 Hz indicating a double bond with *trans* configuration. Signals for three aromatic protons with a characteristic coupling pattern suggested *meta* and *para* substitution in relation to the side chain. Characteristic for the borneol moiety were the three -CH_3_ singlets at 0.90, 0.95 and 0.99 ppm respectively. ESIMS data confirmed the assumed ester structure with two hydroxyl groups as substituents on the aromatic ring by giving a mass of *m/z* 315 [M−H]^−^. The obtained spectroscopic data are in accordance to literature data [5,6,7] for caffeic acid bornyl ester. Similar bornyl esters have been found in *Conocephalum conicum* (liverwort) [8,9] and some *Verbesina* species [7,10,11].

### 2.2. Chemistry

**Scheme 1:**
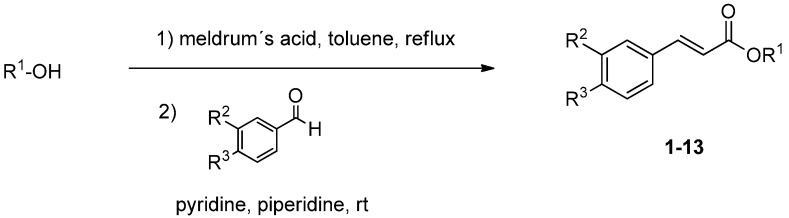
Preparation of compounds **1**–**13** (substituents are listed in Table 1).

Since the caffeoyl-skeleton has been described to be a valuable pharmacophore for antiviral [5,12], antibacterial [13] and antiprotozoal activity [14] we tried to enhance the antileishmanial activity and to decrease the cytotoxicity by the synthesis of a library of derivatives of **1** with systematic structure variations with regard to the terpenoid part on the one hand and the caffeic acid part on the other hand. To investigate structure-activity relationship borneol was replaced by moieties varying in size and bulkiness, and substituents with different properties such as hydroxyl, methoxy and nitro groups as well as halogen atoms were attached to the aromatic ring in varying positions. The general synthesis of phenolic compound **1** and its derivatives **2**–**13** via Knoevenagel-Doebner condensation is outlined in Scheme 1.

**Scheme 2:**
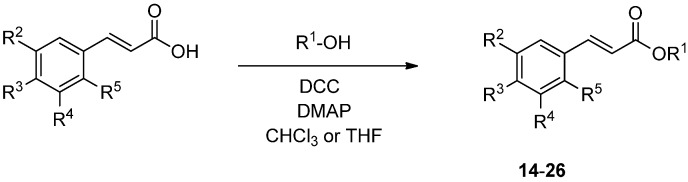
Preparation of compounds **14**–**26** (substituents are listed in Table 2).

Activation of the corresponding alcohol with Meldrum’s acid without isolation of the activated product and subsequent aldol condensation using the substituted aldehyde lead to the cinnamic acid derivatives **1**–**13** in acceptable yields [5]. Compounds **14**–**26** were accessible by facile esterification using the Steglich concept composed of *N*,*N*'-dicyclohexylcarbodiimide (DCC) and 4-dimethyl-aminopyridine (DMAP) in CHCl_3_ or THF (Scheme 2). 

**Scheme 3:**
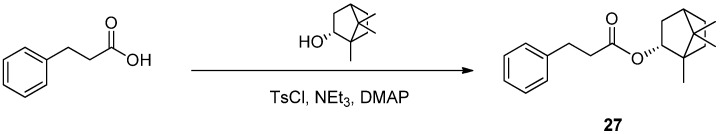
Preparation of compound **27**.

The α,β-unsaturated carbonyl moiety of the caffeic acid is a Michael acceptor which is highly reactive to nucleophilic attacks, e.g., by amino or thiol groups. To investigate whether the double bond is essential for antileishmanial activity compound **27** has been synthesized via a different synthesis route as described before [15]. By esterification of 3-phenyl propanoic acid with borneol using tosyl chloride, Et_3_N and DMAP as a catalyst (Scheme 3) product **27** was obtained.

**Scheme 4:**
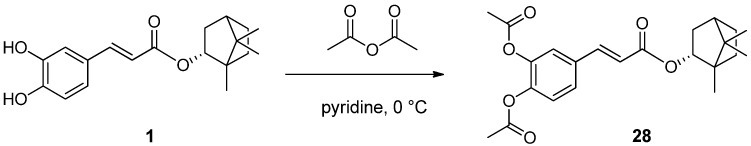
Preparation of compound **28**.

It might be possible that the catechol moiety of compound **1** contributes to cytotoxicity or antileishmanial activity. Hence both phenolic hydroxyl groups were acetylated by adding acetic acid anhydride to caffeic acid bornyl ester **1** (isolated from the plant) in pyridine at 0 °C and stirring for 24 h at room temperature (Scheme 4) to give compound **28**.

### 2.3. Structure-Activity Relationship Investigation

Antileishmanial activities of the synthesized compounds were evaluated in *L. major* promastigotes as described before [16] and in *L. donovani* promastigotes according to Hazra *et al.* [17]. Cytotoxicity was determined on a J774.1 murine cell line [16]. The antiprotozoal activities are presented in Table 1, Table 2 and Table 3 as inhibitory concentrations (IC_50_). The most active compound is the cinnamic acid bornyl ester **15** having an IC_50_ value of 39.6 µM against *L. major* and 15.6 µM against *L. donovani* promastigotes, which is in the concentration range of pentamidine and miltefosine; both are drugs currently in use.

#### 2.3.1. Influence of the Bornyl Moiety

The bornyl moiety is of particular importance of preserving antileishmanial activity. The most active compounds **1**, **7**, **15** and **27** are all esters of borneol. There is no significant change in activity using isoborneol (**1**) instead of borneol (**7**). Substituting borneol with a less sterically demanding alcohol e.g., cyclohexanol (**23**) or eugenol (**24**) gives IC_50_ values higher than 100 µM (*L. major*). Using thymol (**10**, **11**, **19**), menthol (**12**, **13**, **20**) or other bulky substituents like naphthol (**22**) or adamantol (**21**) preserves the activity. A similar pattern is found with *L. donovani* promastigotes. Again the bornyl esters show the best IC_50_ values, and the activity is at a similar level using thymol, menthol or naphthol. In contrast to *L. major* some of the sterically less demanding compounds exhibit activity against *L. donovani* promastigotes (e.g., 23.4 μM (**23**), 41.9 μM (**26**)). This might be due to biological differences between the two species. However borneol seems to be the most advantageous substituent fulfilling the requirement of bulkiness in this position.

#### 2.3.2. The Catechol Moiety

The caffeic acid derivative (**1**) isolated from *V. wallichii* having two hydroxyl groups in position 3 and 4 of the aromatic ring shows an IC_50_ value of 48.8 μM against *L. major* promastigotes and relatively high cytotoxicity (8.3 μM) against a J774.1 cell line. The toxicity is an attribute of all *meta* and *para* dihydroxylated cinnamic acid derivatives (**7**, **10**, **12**) and has been reported in the literature for similar compounds [14,18,19]. The catechol moiety is prone to oxidation resulting in an *o*-quinone which can easily react with amino groups of proteins [20]. Hence, omitting the hydroxyl groups lead to **15** with antileishmanial activity in the same concentration range as **1** but with a fivefold lower cytotoxicity.

**Table 1:** Antileishmanial activity and cytotoxicity (compounds **1**–**13**). 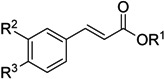

					IC_50_ (μM) *		
Compd.		R^1^	R^2^	R^3^	*L. major* promastigotes	*L. donovani* promastigotes	J774.1
	**1**	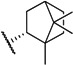	-OH	-OH	48.8	27.3	8.3
	**2**		-OH	-OCH_3_	64.4	41.3	48.7
	**3**		-H	-Cl	71.2	>100	49.5
	**4**		-H	-Br	>100	>100	54.6
	**5**		-H	-N(CH_3_)_2_	>100	>100	>100
	**6**		-H	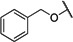	>100	>100	>100
	**7**	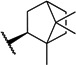	-OH	-OH	45.8	34.8	8.8
	**8**		-OH	-OCH_3_	60.6	74.5	44.3
	**9**		-H	-NO_2_	>100	>100	>100
	**10**	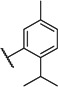	-OH	-OH	57.6	42.1	9.5
	**11**		-OH	-OCH_3_	59.8	79.6	45.6
	**12**	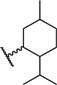	-OH	-OH	59.5	79.4	1.95
	**13**		-OH	-OCH_3_	54.2	>100	44.6

* Positive control: pentamidine 82 μM (*L. major*), 38.6 μM (J774.1); miltefosine: 36.2 μM (*L. major*), 56.5 μM (J774.1); amphotericin B 0.4 μM (*L*. *donovani*).

**Table 2:** Antileishmanial activity and cytotoxicity (compounds **14**–**26** and **28**). 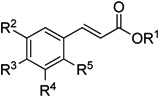

						IC_50_ (µM) *		
Compd.	R^1^	R^2^	R^3^	R^4^	R^5^	*L. major* promastigotes	*L. donovani* promastigotes	J774.1
**14**	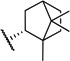	-H	-H	-Cl	-Cl	65.6	>100	58.9
**15**		-H	-H	-H	-H	39.6	15.6	45.2
**16**		-H	-OCH_3_	-H	-H	60.9	>100	42.6
**17**		-H	-Cl	-H	-Cl	80.5	>100	60.6
**18**	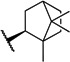	-H	-H	-H	-H	64.3	51.2	46.0
**19**	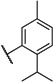	-H	-H	-H	-H	55.0	>100	44.8
**20**	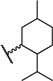	-H	-H	-H	-H	53.1	79.7	44.8
**21**	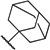	-H	-H	-H	-H	60.0	>100	46.7
**22**	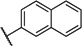	-H	-H	-H	-H	67.0	28.7	44.5
**23**	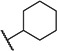	-H	-H	-H	-H	>100	23.4	>100
**24**	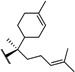	-H	-H	-H	-H	>100	>100	>100
**25**	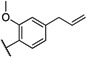	-H	-H	-H	-H	>100	80.3	32.2
**26**	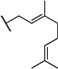	-H	-H	-H	-H	>100	41.9	>100
**28**	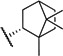	-OAc	-OAc	-H	-H	30.7	-	2.1

* Positive control: pentamidine 82 μM (*L. major*), 38.6 μM (J774.1); miltefosine: 36.2 μM (*L. major*), 56.5 μM (J774.1); amphotericin B 0.4 μM (*L*. *donovani*).

The same effect is observed by replacement of the hydroxyl group in 4-position with a methoxy group (compounds **2**, **8**, **11**, **13**). However, the cytotoxicity is not correlating with antileishmanial activity, the latter stays in the same concentration range for compounds whether with or without hydroxyl groups. Acetylation to “mask” the catechol structure (compound **28**) slightly increased antileishmanial activity but cytotoxicity persisted in the single-digit micromolar range.

#### 2.3.3. Substitution on the Aromatic Ring

In order to analyse the influence of the substituents of the aromatic ring on antileishmanial activity and cytotoxicity, bornyl esters are compared in the following. Compound **15** with an unsubstituted benzene ring is the most promising compound with an antileishmanial activity in a low micromolar concentration and low cytotoxicity. The comparison of all derivatives with substituents in *para* position reveals compounds **3** (-Cl) and **16** (-OMe) to have minor antileishmanial activity against *L. major* (71.2 μM and 60.9 μM). Larger substituents are not tolerated in this location (compounds **4**–**6**, **9**) whereas chlorination in position 2 and 3 (compound **14**), and 2 and 4 (compound **17**), respectively, is acceptable. None of these compounds exhibited any activity against *L. donovani* promastigotes. Thus, a non-substituted aromatic ring is advantageous for a good antiprotozoal activity against both species.

#### 2.3.4. Michael System

All compounds are characterized by the highly reactive enone Michael system, which is prone to unspecific covalent reactions with proteins of both parasite and host. In order to check whether the Michael moiety is necessary for antileishmanial activity, compound **27** characterized by a simple carbonyl group was synthesized. This compound is as active as the corresponding compound **15** and did not show cytotoxicity (Table 3). This is in contrast to the results observed for caffeic acid alkyl ester derivatives which were found to be inactive without the double bond [14].

**Table 3:** Antileishmanial activity of compounds **15** and **27**.

Compound	*L. major* promastigotes IC_50_ (µM)	Cytotoxicity J774.1 IC_50_ (µM)	Selectivity index SI *^a^*
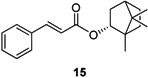	39.6	45.2	1.1
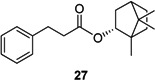	50.2	>100	>2.0

*^a^* SI = IC_50_ for J774.1/IC_50_ for *L*. *major*.

### 2.4. Amastigote Results

The amastigote is the intracellular pathogenic form of the parasite in the vertebrate host and therefore the important target. Compounds **1**, **2**, **7**, **10**, **15**, **19**, **20** showing high activity against *L. major* promastigotes were selected for screening against *L. major* amastigotes and cytotoxicity against BMDM. The amastigote assay was conducted as reported by Bringmann *et al.* [16] and the results are presented in Table 4. The cytotoxicity against BMDM mirrors those against the macrophages J774.1. With regard to antileishmanial activity cinnamic acid menthyl (**20**) and thymyl ester (**19**) show good activities combined with low cytotoxicity. Again the cinnamic acid bornyl ester (**15**) is the most active compound. In general, the activity lies in the range of miltefosine with an IC_50_ value of 33.0 μM. Based on these results, the compounds should be tested for *in vivo* activity against *Leishmania* infections.

**Table 4:** Antileishmanial activity and cytotoxicity of selected compounds.

Compound	*L.major* amastigotes IC_50_ (µM)	Cytotoxicity BMDM IC_50_ (µM)	Selectivity index SI *^a^*
**1**	47.6	5.3	0.1
**7**	47.0	10.9	0.2
**15**	10.9	54.3	5.0
**2**	39.2	49.0	1.2
**19**	49.1	>148	>3.0
**20**	19.5	>126	>6.4
**10**	54.1	9.8	0.2
**Miltefosine**	33.0	65.5	2.0

*^a^* SI = IC_50_ for BMDM/IC_50_ for *L*. *major*.

## 3. Experimental

### 3.1. General Procedures

Starting materials and reagents were purchased from Sigma-Aldrich (Taufkirchen, Germany) and VWR (Darmstadt, Germany). Solvents were of synthetic or analytical grade. Melting points were determined on a Stuart melting point apparatus SMP10 (Bibby Scientific, Stone, United Kingdom) and are uncorrected. Optical rotations were measured on a CHIRALYSER 6.3 (IBZ Messtechnik, Springe, Germany). IR spectra were acquired on a JASCO (Gross-Umstadt, Germany) FT/IR-6100 Fourier Transformation Infrared Spectrometer equipped with an ATR unit. ^1^H (400.132 MHz) and ^13^C (100.613 MHz) NMR spectra were recorded on a Bruker Avance 400 Ultra Shield™ (Bruker Biospin, Ettlingen, Germany) spectrometer. The signals of the deuterated solvents were used as internal standards (CDCl_3_: ^1^H 7.26 ppm, ^13^C 77.0 ppm; MeOD: ^1^H 4.84 ppm, ^13^C 49.05 ppm). LC/MS was conducted on an Agilent 1100 analytical HPLC with DAD detection and an Agilent LC/MSD Trap (Agilent Technologies, Böblingen, Germany). ESIMS data was conducted in positive and negative mode. For LC/MS, following conditions were used: Nucleodur Sphinx RP-18 (Macherey-Nagel, Düren, Germany, 150 mm × 4.6 mm, 5 µm, A) CH_3_CN + 0.1% FA, B) H_2_O + 0.1% formic acid (FA), gradient: 10% B (0–2 min), 30% B (2–3 min), 100% B (3–25 min), 80% B (25–33 min), 40% B (33–37 min), 10% B (38–40 min), 0.8 mL/min, MS-detection: ESI, nebulizer pressure: 50 psi, drying gas: 10 L/min, drying gas temperature: 350 °C, capillary voltage: 3500 V. An Agilent 1100 preparative HPLC with fraction collector and multiple wavelength detector (MWD) was used for fractionation of the extract.

### 3.2. Plant Material and Preparation of Extract

The plant material was obtained as described previously [4]. The rhizomes were pulverized and a portion of 10 g was refluxed for 2 h with 100 mL chloroform. Evaporation of the solvent yielded a black syrup with a characteristic smell.

### 3.3. Bioactivity-Guided Fractionation and Isolation of **1**

**Figure 1:**
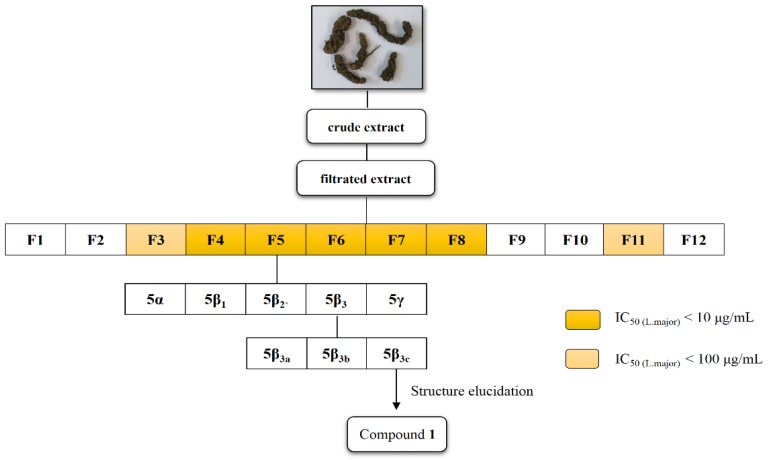
Overview of bioactivity-guided fractionation.

The crude chloroform extract (10.9 g) was subjected to a bioactivity-guided fractionation (Figure 1). The extract was slurried in MeOH and the insoluble parts were removed by filtration over LiChroprep^©^ RP-18 material (Yield: 9.5 g).

The extract was subjected to semi-preparative HPLC (250 mm × 10 mm, 5 µm, Macherey-Nagel Nucleosil 100-5; (A) H_2_O, (B) MeOH, gradient: 70% B (0 min), 75% B (7 min), 100% B (25 min), 70% B (30 min); flow rate: 3.3 mL/min) and partitioned into 12 fractions. Fraction 4–8 exhibited significant antileishmanial activity against *L. major* promastigotes. Fraction 5 (559 mg) was further fractionated by column chromatography using silica gel and MeOH/CHCl_3_ (4.8:0.2 *v/v*) as mobile phase. Fraction 5β_3_ (258 mg) was partitioned a second time by column chromatography using silica gel and MeOH/CHCl_3_ (4.8:0.2 *v/v*) to yield three fractions (5β_3a_, 5β_3b_, 5β_3c_) from which the last fraction afforded 135 mg of caffeic acid (−)-bornyl ester (IC_50_ = 48.8 µM). The structure was identified by means of NMR and LC/MS data.

*Caffeic Acid (−)-bornyl Ester* (**1**)*.* Brownish solid (MeOH); m.p. 150 °C (lit.[6] 148–151 °C); [α]_426_ −5.1° (c 0.1, MeOH); IR: 3444, 3165, 1661, 1604, 1273, 1186 cm^−1^; ^1^H-NMR (MeOD) *δ* (ppm) *J* (Hz): 0.90 (s; 3H); 0.94 (s; 3H); 0.98 (s; 3H); 1.06 (dd; *3.7*; *13.7*; 1H); 1.26–1.35 (m;2H); 1.70 (t; *4.5*; 1H); 1.79–1.88 (m; 1H); 2.07–2.13 (m; 1H); 2.38–2.46 (m; 1H); 5.00 (ddd; *2.2*; *3.4*; *9.9*; 1H); 6.30 (d; *15.9*; 1H); 6.80 (d; *8.2*; 1H); 7.00 (dd; *2.1*; *8.2*; 1H); 7.05 (d; *2.1*; 1H); 7.54 (d; *15.9*; 1H); ^13^C-NMR (MeOD) *δ* (ppm) 13.9; 19.2; 20.1; 28.2; 29.0; 37.9; 46.4; 48.9; 49.5; 81.2; 115.1; 115.5; 116.5; 122.9; 127.8; 146.7; 146.8; 149.6; 169.7; ESIMS: *m/z* 315 [M−H]^−^, 339 [M+Na]^+^.

### 3.4. Synthesis

#### 3.4.1. General Procedure for the Synthesis of Compounds **1**–**13**

Synthesis was conducted according to the method of Xia *et al.* [5]. The alcohol (2.94 mmol) and Meldrum’s acid (2.94 mmol) were dissolved in toluene (20 mL) and refluxed for 4 h. To the resulting activated alcohol the corresponding aldehyde (2.94 mmol), pyridine (2.5 mL) and piperidine (250 µL) were added. This mixture was stirred at room temperature for 24–48 h. The solvent was evaporated, the residue dissolved in diethyl ether (20 mL) and washed three times with an aqueous saturated NaHCO_3_ solution (10 mL), 20% HCl (10 mL), and H_2_O (10 mL), respectively. After drying over MgSO_4_ the solvent was evaporated and the residue was chromatographed using silica gel (petroleum ether/ethyl acetate 60:40) to yield the desired product (overall yields of the two steps are reported).

*Synthesized Caffeic Acid (−)-bornyl Ester* (**1**)*.* Yield 19%; off-white solid; m.p. 150 °C; [α]_426_ −4.5° (c 0.1, MeOH); IR and NMR data in accordance with above and literature [6].

*Isoferulic Acid (−)-bornyl Ester* (**2**)*.* Yield 9%; colorless syrup; IR: 3384, 2952, 2877, 1698, 1631, 1263, 1172, 1155 cm^−1^; ^1^H-NMR (CDCl_3_) *δ* (ppm) *J* (Hz): 0.88 (s; 3H); 0.90 (s; 3H); 0.95 (s; 3H); 1.04–1.08 (m; 1H); 1.25–1.32 (m; 2H); 1.71 (t *4.5*; 1H); 1.75–1.85 (m; 1H); 2.02–2.09 (m; 1H); 2.38–2.46 (m; 1H); 3.94 (s; 3H); 5.02 (ddd; *2.1*; *3.4*; *9.9*; 1H); 6.30 (d; *16.0*; 1H); 6.92 (d; *8.2*; 1H); 7.05 (d; *1.9*; 1H); 7.09 (dd; *8.2*; *1.9*; 1H); 7.60 (d; *16.0*; 1H); ^13^C-NMR (CDCl_3_) *δ* (ppm): 13.6; 18.9; 19.7; 27.3; 28.1; 36.9; 45.0; 47.9; 48.9; 56.0; 79.8; 109.3; 114.7; 116.2; 123.0; 127.1; 144.3; 146.7; 147.8; 167.6.

*4-Chlorocinnamic Acid (−)-bornyl Ester* (**3**). Yield 14%; slightly yellow solid; m.p. 80–83 °C; IR: 2952, 2876, 1704, 1636, 1490, 1308, 1184, 820 cm^−1^; ^1^H-NMR (CDCl_3_) *δ* (ppm) *J* (Hz): 0.88 (s; 3H); 0.90 (s; 3H); 0.94 (s; 3H); 1.03–1.08 (m; 1H); 1.25–1.32 (m; 2H); 1.71 (t; *4.6*; 1H); 1.74–1.84 (m; 1H); 2.01–2.06 (m; 1H); 2,38–2.46 (m; 1H); 5.02 (ddd; *2.2*; *3.4*; *10.0*; H); 6.44 (d; *16.0*; 1H); 7.36 (d; *8.5*; 2H); 7.47 (d; *8.5*; 2H); 7.61 (d; *16.0*; 1H); ^13^C-NMR (CDCl_3_) *δ* (ppm): 13.5; 18.9; 19.7; 27.2; 28.1; 36.9; 45.0; 47.9; 49.0; 80.2; 119.4; 129.1 (2C); 129.2 (2C); 133.1; 136.0; 142.7; 167.1.

*4-Bromocinnamic Acid (−)-bornyl Ester* (**4**). Yield 9%; yellow solid; m.p. 71–74 °C; IR: 2953, 2875, 1705, 1635, 1203, 1158, 816 cm^−1^; ^1^H-NMR (CDCl_3_) *δ* (ppm) *J* (Hz): 0.88 (s;3H); 0.90 (s;3H); 0.94 (s; 3H); 1.03–1.08 (m; 1H); 1.25–1.32 (m;2H); 1.71 (t; *4.6*; 1H); 1.74–1.84 (m;1H); 2.01–2.06 (m;1H); 2.38–2.46 (m; 1H); 5.03 (ddd; *2.2*; *3.4*; *9.9*; 1H); 6.44 (d; *16.0*; 1H); 7.36 (d; *8.5*; 2H); 7.47 (d; *8.5*; 2H); 7.61 (d; *16.0*; 1H); ^13^C-NMR (CDCl_3_) *δ* (ppm): 13.5; 18.9; 19.7; 27.2; 28.1; 36.9; 45.0; 47.9; 48.9; 80.2; 119.4; 124.4; 129.4 (2C); 132.1 (2C); 133.4; 142.7; 167.1.

*4-N-Dimethylaminocinnamic Acid (−)-bornyl Ester* (**5**). Yield 25%; yellowish solid; m.p. 114–116 °C; IR: 2950, 2877, 1696, 1603, 1524, 1151, 810 cm^−1^; ^1^H-NMR (CDCl_3_) *δ* (ppm) *J* (Hz): 0.88 (s; 3H); 0.90 (s; 3H); 0.94 (s; 3H); 1.04–1.08 (m; 1H); 1.25–1.37 (m; 2H); 1.70 (t; *4.5*; 1H); 1.74–1.84 (m; 1H); 2.03–2.10 (m; 1H); 2.37–2.45 (m; 1H); 3.02 (s; 6H); 5.01 (ddd; *2.0*; *3.3*; *9.9*; 1H); 6.25 (d; *15.9*; 1H); 6.67 (d; *8.8*; 2H); 7.43 (d; *8.8*; 2H); 7.61 (d; *15.9*; 1H); ^13^C-NMR (CDCl_3_) *δ* (ppm): 13.6; 18.9; 19.8; 27.3; 28.1; 36.9; 40.1 (2C); 45.0; 47.8; 48.9; 79.4; 111.8 (2C); 113.2; 123.0; 129.7 (2C); 144.7; 151.7; 168.2.

*4-O-Benzoylcinnamic Acid (−)-bornyl Ester* (**6**)*.* Yield 13%; lightly yellow solid; m.p. 93–95 °C; IR: 2951, 2878, 1695, 1627, 1600, 1510, 1255, 1171, 998 cm^−1^; ^1^H-NMR (CDCl_3_) *δ* (ppm) *J* (Hz): 0.88 (s; 3H); 0.90 (s; 3H); 0.95 (s; 3H); 1.04–1.08 (m; 1H); 1.25–1.37 (m; 2H); 1.70 (t; *4.5*; 1H); 1.73–1.84 (m; 1H); 2.02–2.08 (m; 1H); 2.37–2.45 (m; 1H); 5.01 (ddd; *2.0*; *3.4*; *9.9*; 1H); 5,10 (s; 2H); 6.34 (d; *16.0*; 1H); 7.00 (d; *8.7*; 2H); 7.34–7.44 (m; 5H); 7.49 (d; *8.7*; 2H); 7.60 (d; *16.0*; 1H); ^13^C-NMR (CDCl_3_) *δ* (ppm): 13.5; 18.9; 19.7; 27.2; 28.1; 36.9; 45.0; 47.8; 48.9; 70.1; 79.8; 115.2 (2C); 116.5; 127.4 (2C); 127.5; 128.0; 128.7 (2C); 129.7 (2C); 136.5; 143.8; 160.4; 167.6.

*Caffeic Acid Isobornyl Ester* (**7**). Yield 12%; brownish solid; m.p. 160–161 °C; IR: 3444, 3168, 2956, 1666, 1604, 1439, 1277, 1182 cm^−1^; ^1^H-NMR (CDCl_3_) *δ* (ppm) *J* (Hz): 0.86 (s; 3H); 0.89 (s;3H); 1.05 (s; 3H); 1.09–1.25 (m; 2H); 1.55–1.62 (m; 1H); 1.68–1.88 (m; 4H); 4.80 (dd; *4.6*; *6.9*; 1H); 6.23 (d; *15.9*; 1H); 6.87 (d; *8.2*; 1H); 6.99 (dd; 1.7; *8.2*; 1H); 7.12 (d; *1.7*; 1H); 7.53 (d; *15.9*; 1H); ^13^C-NMR (CDCl_3_) *δ* (ppm) 11.2; 19.7; 19.8; 26.7; 33.4; 38.5; 44.8; 46.7; 48.6; 81.2; 114.1; 115.2; 115.7; 122.1; 127.1; 143.6; 144.5; 146.3; 167.4.

*Isoferulic Acid Isobornyl Ester* (**8**). Yield 11%; colourless syrup; IR: 3387, 2952, 2876, 1696, 1631, 1591, 1512, 1263, 1154 cm^−1^; ^1^H-NMR (CDCl_3_) *δ* (ppm) *J* (Hz): 0.87 (s;3H); 0.90 (s;3H); 1.07 (s;3H); 1.09–1.26 (m;2H); 1.54–1.61 (m;1H); 1.68–1.90 (m;4H); 3.94 (s;3H); 4.80 (dd;*4.3*;*7.4*;1H); 6.25 (d;*15.9*;1H); 6.91 (d;*8.2*;1H); 7.02 (d;*1.9*;1H); 7.07 (dd;*1.9*;*8.2*;1H); 7.55 (d;*15.9*;1H); ^13^C-NMR (CDCl_3_) *δ* (ppm) 11.5; 20.0; 20.1; 27.0; 33.8; 38.9; 45.1; 47.0; 48.9; 56.0; 81.0; 109.3; 114.7; 116.3; 123.0; 127.1; 144.3; 146.8; 147.8; 167.0.

*4-Nitrocinnamic Acid Isobornyl Ester* (**9**). Yield 16%; yellowish solid; m.p. 121–123 °C; IR: 2950, 2876, 1700, 1516, 1340, 1301, 1163, 842 cm^−1^; ^1^H-NMR (CDCl_3_) *δ* (ppm) *J* (Hz): 0.87 (s; 3H); 0.90 (s; 3H); 1.06 (s; 3H); 1.07–1.25 (m; 2H); 1.54–1.61 (m; 1H); 1.70–1.92 (m; 4H); 4.83 (dd; *4.1*; *7.5*; 1H); 6.53 (d; *16.0*; 1H); 7.65 (d; *16.0*; 1H); 7.67 (d; *8,7*; 2H); 8.24 (d; *8.7*; 2H); ^13^C-NMR (CDCl_3_) *δ* (ppm) 11.5; 20.0; 20.1; 27.0; 33.8; 38.8; 45.1; 47.0; 49.0; 81.8; 123.2; 124.1 (2C); 128.6 (2C); 140.7; 141.3; 165.6.

*Caffeic Acid Thymyl Ester* (**10**). Yield 14%; brownish solid; m.p. 115–117 °C; IR: 3339, 2960, 1726, 1695, 1616, 1514, 1235, 1136, 1114 cm^−1^; ^1^H-NMR (CDCl_3_) *δ* (ppm) *J* (Hz): 1.20 (s; 3H); 1.21 (s; 3H); 2.32 (s; 3H); 3.03 (sept; *6.9*; 1H); 5.84 (br s; -OH); 5.89 (br s; -OH); 6.48 (d; *15.9*; 1H); 6.87 (d; *8.2*; 1H); 6.87 (d; *0.9*; 1H); 7.04 (dd; *0.9*; *7.9*; 1H); 7.07 (dd; *2.0*; *8.2*; 1H); 7.22 (d; *7.9*; 1H); 7.77 (d; *15.9*; 1H); ^13^C-NMR (CDCl_3_) *δ* (ppm) 20.5; 22.7 (2C); 26.9; 114.2; 114.4; 115.2; 122.4; 122.5; 126.2; 126.9; 127.0; 136.3; 136.9; 143.5; 146.3; 146.4; 147.6; 166.2.

*Isoferulic Acid Thymyl Ester* (**11**). Yield 15%; colourless syrup; IR according to ref. [21]; ^1^H-NMR (CDCl_3_) *δ* (ppm) *J* (Hz): 1.21 (s; 3H); 1.23 (s; 3H); 2.32 (s; 3H); 2.34 (s; 3H); 3.05 (sept; *6.9*; 1H); 3.96 (s; 3H); 6.52 (d; *15.9*; 1H); 6.89 (d; *0.9*; 1H); 6.96 (d; *8.2*; 1H); 7.05 (dd; *0.9*; *7.9*; 1H); 7.11 (d; *1.9*; 1H); 7.12 (dd; *1.9*; *8.2*; 1H); 7.23 (d; *7.9*; 1H); 7.81 (d; *15.9*; 1H); ^13^C-NMR (CDCl_3_) *δ* (ppm) 21.2; 23.4 (2C); 27.5; 56.3; 109.8; 114.9; 115.1; 123.2; 123.7; 126.7; 127.1; 127.4; 136.8; 137.5; 146.8; 147.1; 148.3; 148.6; 166.3.

*Caffeic Acid Menthyl Ester* (**12**). Yield 25%; colourless syrup; IR: 3384, 2953, 2868, 1694, 1632, 1591, 1511, 1264, 1170 cm^−1^; ^1^H-NMR (CDCl_3_) *δ* (ppm) *J* (Hz): 0.78 (d; *6.9*; 3H); 0.90 (d; *2.7*; 3H); 0.90 (d; *2.7*; 3H); 1.00–1.15 (m; 2H); 1.42–1.55 (m; 2H); 1.69–1.72 (m; 2H); 1.87–1.95 (m; 1H); 2.03–2.06 (m; 1H); 4.8 (dt; *4.4*; *10.9*; 1H); 6.25 (d; *15.9*; 1H); 6.87 (d; *8.2*; 1H); 6.99 (dd; *1.9*; *8.2*; 1H); 7.11 (d; *1.9*; 1H); 7.57 (d; *15.9*; 1H); ^13^C-NMR (CDCl_3_) *δ* (ppm) 16.8; 21.1; 22.4; 24.0; 26.8; 31.8; 34.7; 41.4; 47.6; 75.1; 114.8; 115.8; 116.2; 122.7; 127.8; 144.4; 145.4; 147.0; 168.2.

*Isoferulic Acid Menthyl Ester* (**13**). Yield 24%; colourless syrup; IR: 3398, 2953, 2926, 2868, 1695, 1513, 1263, 1158 cm^−1^; ^1^H-NMR (CDCl_3_) *δ* (ppm) *J* (Hz) 0.79 (d; *6.9*; 3H); 0.91 (d; *2.9*; 3H); 0.92 (d; *2.6*; 3H); 1.00–1.16 (m; 2H); 1.40–1.55 (m; 2H); 1.67–1.73 (m; 2H); 1.90–1.99 (m; 1H); 2.03–2.09 (m; 1H); 3.93 (s; 3H); 4.82 (dt; *4.4*; *10.9*; 1H); 6.28 (d; *15.8*; 1H); 6.91 (d; *8.2*; 1H); 7.04 (d; *1.8*; 1H); 7.07 (dd; *1.8*; *8.2*; 1H); 7.60 (d; *15.8*; 1H); ^13^C-NMR (CDCl_3_) *δ* (ppm) 16.4; 20.8; 22.0; 23.6; 26.3; 31.4; 34.3; 41.1; 47.3; 55.9; 74.1; 109.2; 114.7; 116.1; 123.0; 127.1; 144.4; 146.7; 147.8; 166.8.

#### 3.4.2. General Procedure for the Synthesis of Compounds **14**–**27**

Cinnamic acid (500 mg, 3.37 mmol) and the respective alcohol (3.37 mmol) were dissolved in THF or CHCl_3_ (20 mL). After addition of DCC and DMAP the resulting mixture was stirred for 16 h at room temperature. After evaporation and column chromatography on silica gel (CHCl_3_/MeOH 4.8:0.2) the desired product was obtained.

*2,3-Dichlorocinnamic acid (−)-bornyl ester* (**14**). Yield 18%; colourless crystals; m.p. 86–87 °C; IR: 2930, 2875, 2118, 1711, 1635, 1315, 1178 cm^−1^; ^1^H-NMR (CDCl_3_) *δ* (ppm) *J* (Hz) 0.89 (s; 3H); 0.90 (s; 3H); 0.95 (s; 3H); 1.05–1.09 (m; 1H); 1.25–1.38 (m; 2H); 1.72 (t; *4.5*; 1H); 1.75–1.83 (m; 1H); 2.00–2.06 (m; 1H); 2.39–2.47 (m; 1H); 5.03 (ddd; *2.2*; *3.4*; *9.9*; 1H); 6.44 (d; *16.0*; 1H); 7.22 (t; *7.9*; 1H); 7.48 (dd; *1.5*; *7.9*; 1H); 7.54 (dd; *1.5*; *7.9*; 1H); 8.09 (d; *16.0*; 1H); ^13^C-NMR (CDCl_3_) *δ* (ppm) 13.2; 18.6; 19.4; 26.9; 27.7; 36.5; 44.6; 47.6; 48.6; 80.1; 122.3; 125.5; 127.0; 131.1; 132.6; 133.7; 134.9; 139.7; 166.2.

*Cinnamic Acid (−)-Bornyl Ester* (**15**). Yield 7%; colourless syrup; spectral data are in accordance with data reported in ref. [22,23].

*4-Methoxycinnamic Acid (−)-Bornyl Ester* (**16**). Yield 25%; crystalline solid; m.p. 87–90 °C; IR: 2952, 2929, 2117, 1701, 1627, 1602, 1513, 1152 cm^−1^; ^1^H-NMR (CDCl_3_) *δ* (ppm) *J* (Hz) 0.87 (s; 3H); 0.88 (s; 3H); 0.94 (s; 3H); 1.03–1.08 (m; 1H); 1.24–1.39 (m; 2H); 1.70 (t; *4.5*; 1H); 1.73–1.80 (m; 1H); 2.02–2.08 (m; 1H); 2.37–2.45 (m; 1H); 3.84 (s; 3H); 5.02 (ddd; *2.2*; *3.4*; *9.9*; 1H); 6.34 (d; *15.9*; 1H); 6.90 (d; *8.7*; 2H); 7.49 (d; *8.7*; 2H); 7.63 (d; *15.9*; 1H); ^13^C-NMR (CDCl_3_) *δ* (ppm) 13.2; 18.6; 19.4; 26.9; 27.7; 36.6; 44.7; 47.5; 48.6; 55.0; 79.4; 114.0 (2C); 116.0; 127.0; 129.3 (2C); 143.5; 160.9; 167.3.

*2,4-Dichlorocinnamic Acid (−)-bornyl Ester* (**17**). Yield 85%; colourless crystalline solid; m.p. 90–92 °C; IR: 2930, 2882, 2118, 1712, 1638, 1469, 1312, 1177 cm^−1^; ^1^H-NMR (CDCl_3_) *δ* (ppm) *J* (Hz): 0.90 (s; 3H); 0.92 (s; 3H); 0.97 (s; 3H); 1.07–1.11 (m; 1H); 1.27–1.40 (m; 2H); 1.74 (t; *4.5*; 1H); 1.76–1.85 (m; 1H); 2.02–2.09 (m; 1H); 2.41–2.49 (m; 1H); 5.03 (ddd; *2.2*; *3.4*; *9.9*; 1H); 6.44 (d; *16.0*; 1H); 7.22 (t; *7.9*; 1H); 7.48 (dd; *1.5*; *7.9*; 1H); 7.54 (dd; *1.5*; *7.9*; 1H); 8.09 (d; *16.0*; 1H); ^13^C-NMR (CDCl_3_) *δ* (ppm) 13.5; 18.9; 19.7; 27.2; 28.0; 36.8; 44.9; 47.9; 49.0; 80.4; 121.8; 127.5; 128.3; 130.0; 131.4; 135.5; 136.2; 138.8; 166.5.

*Cinnamic Acid Isobornyl Ester* (**18**). Yield 14%; colourless syrup; IR: 2952, 2877, 1708, 1637, 1309, 1160 cm^−1^; ^1^H-NMR (CDCl_3_) *δ* (ppm) *J* (Hz) 0.88 (s; 3H); 0.90 (s; 3H); 1.07 (s; 3H); 1.09–1.24 (m; 2H); 1.55–1.62 (m; 1H); 1.68–1.91 (m; 4H); 4.81 (dd; *4.3*; *7.3*; 1H); 6.41 (d; *16.0*; 1H); 7.36–7.40 (m; 3H); 7.00–7.54 (m; 2H); 7.63 (d; *16.0*; 1H); ^13^C-NMR (CDCl_3_) *δ* (ppm) 11.5; 20.0; 20.1; 27.0; 33.8; 38.9; 45.1; 47.0; 48.9; 81.1; 118.9; 128.0 (2C); 128.8 (2C); 130.1; 134.5; 144.2; 167.0. 

*Cinnamic Acid Thymyl Ester* (**19**). Yield 24%; colourless solid; m.p. 64 °C; spectral data are in accordance with data reported in ref. [24].

*Cinnamic Acid Menthyl Ester* (**20**). Yield 25%; colourless crystals; m.p. 51–53 °C; spectral data are in accordance with data reported in ref. [25,26,27].

*Cinnamic Acid Adamantyl Ester* (**21**). Yield 10%; colourless powder; m.p. 66 °C [lit. [28]: 63–65 °C]; IR: 2896, 2866, 2848, 1703, 1687, 1641, 1170 cm^−1^; ^1^H-NMR (CDCl_3_) *δ* (ppm) *J* (Hz) 1.70 (m; 6H); 2.20 (s; 9H); 6.36 (d; *15.9*; 1H); 7.36–7.38 (m; 3H); 7.49 (m; 2H); 7.57 (d; *15.9*; 1H); ^13^C-NMR (CDCl_3_) *δ* (ppm) 30.9 (3C); 36.2 (3C); 41.4 (3C); 80.6; 102.4; 128.0; 128.8; 129.9; 134.7; 143.4; 166.0.

*Cinnamic Acid Naphthyl Ester* (**22**). Yield 15%; colourless crystalline solid; m.p. 109–110 °C [lit. [24] 106–107 °C]; spectral data are in accordance with data reported in ref. [24].

*Cinnamic Acid Cyclohexyl Ester* (**23**). Yield 33%; colourless liquid; spectral data are in accordance with data reported in ref. [29,30,31].

*Cinnamic Acid Bisabolyl Ester* (**24**). Yield 11%; yellowish syrup; IR: 2962, 2924, 1703, 1636, 1160 cm^−1^; ^1^H-NMR**(**CDCl_3_) *δ* (ppm) *J* (Hz) 1.28–1.33 (m; 1H); 1.41 (s; 3H); 1.53 (s; 3H); 1.58 (s; 3H); 1.59 (s; 3H); 1.74–2.00 (m; 9H); 5.04 (br m; 1H); 5.31 (br m; 1H); 6.32 (d; *16.0*; 1H); 7.29–7.32 (m; 3H); 7.43–7.46 (m; 2H); 7.52 (d; *16.0*; 1H); ^13^C-NMR (CDCl_3_) *δ* (ppm) 17.6; 20.6; 22.1; 23.3; 23.7; 25.7; 26.4; 30.9; 35.7; 40.5; 87.3; 120.1; 120.3; 124.1; 128.0 (2C); 128.8 (2C); 130.0; 131.6; 134.1; 134.7; 143.5; 166.1.

*Cinnamic Acid Eugenyl Ester* (**25**). Yield 50%; colourless plates; all data are in accordance with data reported in ref. [21,32].

*Cinnamic Acid Geranyl Ester* (**26**). Yield 28%; colourless liquid; spectral data are in accordance with data reported in ref. [33].

#### 3.4.3. Synthesis of Phenyl Propanoic Acid (−)-Bornyl Ester (**27**)

Propanoic acid (700 mg, 4.66 mmol) was dissolved in NEt_3_ (3.2 mL, 5 eq.) and cooled in an ice bath. 4-Dimethylaminopyridine (114 mg, 0.2 eq.) in CH_3_CN (2 mL) and tosyl chloride (1.07 g, 1.2 eq.) in CH_3_CN (3 mL) were added and the solution stirred for 30 min until a red colour developed. Then (−)-borneol (719 mg, 4.66 mmol) dissolved in CH_3_CN (2 mL) was added to the solution. The mixture was allowed to warm up to room temperature and stirred for two hours. After evaporation of the solvent the reaction mixture was suspended in water (10 mL) and extracted three times with diethyl ether (10 mL). The organic phase was washed three times each with water, brine and Na_2_SO_4_. After drying the organic phase over MgSO_4_ the product was purified twice by column chromatography on silica gel using ethyl acetate/petroleum ether (60:40) as eluent to yield 278 mg of **27**. Yield 21%; colourless liquid; IR: 2952, 2877, 1730 cm^−1^; ^1^H-NMR (MeOD) *δ* (ppm) *J* (Hz) 0.78 (s; 3H); 0.89 (s; 3H); 0.91 (s; 3H); 0.84–0.88 (m; 2H); 1.14–1.20 (m; 1H); 1.26–1.34 (m; 1H); 1.63 (t; *4.5*; 1H); 1.72–1.80 (m; 1H); 1.87–1.94 (m; 1H); 2.26–2.34 (m; 1H); 2.67 (t; *7.4*; 2H); 2.95 (t; *7.4*; 2H); 4.84 (m; 1H); 7.11–7.29 (m; 5H); ^13^C-NMR (MeOD) *δ* (ppm) 13.8; 19.2; 20.1; 28.1; 28.9; 32.2; 37.0; 37.7; 46.3; 48.8; 49,7; 81.4; 127.3; 129.4 (2C); 129.5 (2C); 141.8; 175.0.

#### 3.4.4. Synthesis of 3-(3,4-Bis(acetyloxy))phenyl Propenoic Acid (−)-Bornyl Ester (**28**)

Compound **1** (20 mg) was suspended in pyridine (2 mL) at 0 °C. One equivalent of acetic acid anhydride was added and the solution stirred for 2 h at room temperature. After evaporation the residue was subjected to column chromatography on silica gel using CHCl_3_/MeOH (4.8:0.2 *v/v*) as eluent to yield the desired product. Yield 51%; orange syrup; IR: 2953, 2877, 1771, 1707 cm^−1^; ^1^H-NMR (CDCl_3_) *δ* (ppm) *J* (Hz) 0.87 (s; 3H); 0.90 (s; 3H); 0.94 (s; 3H); 1.03–1.07 (m; 1H); 1.25–1.35 (m; 2H); 1.71 (t; *4.5*; 1H); 1.75–1.81 (m; 1H); 1.99–2.05 (m; 1H); 2.30 (s; 3H); 2.31 (s; 3H); 2.37–2.47 (m; 1H); 5.01 (ddd; *2.1*; *3.4*; *9.9*; 1H); 6,41 (d; *16.0*; 1H); 7.22 (d; *8.4*; 1H); 7.38 (d; *2.0*; 1H); 7,42 (dd; *2.0*; *8.4*; 1H); 7.60 (d; *16.0*; 1H); ^13^C-NMR (CDCl_3_) *δ* (ppm) 13.5; 18.9; 19.7; 20.6; 20.7; 27.2; 28.0; 36.8; 45.0; 47.9; 49.0; 80.2; 120.0; 122.7; 123.9; 126.3; 133.5; 142.2; 142.4; 143.4; 166.9; 168.0; 168.1.

### 3.5. Biological Assays

Materials and methods for the biological assays have been described before by the authors. AlamarBlue assays for investigation of antileishmanial activities against *L. major* promastigotes, amastigotes and J774.1 and BMDM cytotoxicity were conducted as previously reported [16]. The protocol for the *L. donovani* quantitative colorimetric assay is outlined by Hazra *et al.* [17]. The IC_50_ values are presented as mean values of two independent experiments against the parasite and macrophages.

## 4. Conclusions

In summary, caffeic acid bornyl ester (**1**) was isolated as the antileishmanial component of the chloroform extract of *V. wallichii* rhizome. Structure-activity relationships of a compound library of 27 derivatives were analysed and revealed the size of the non-cinnamyol part of the molecule to have a significant influence on antileishmanial activity. Hydroxyl groups in the 3 and 4 positions of the aromatic ring increase cytotoxicity and the Michael system in the side chain is not essential for antiprotozoal activity against *L. major* promastigotes (Table 3). The cinnamic acid bornyl ester **15** showed the best activity with regards to *L. major* and *L. donovani* promastigotes with acceptable cytotoxicity. Compound **27** with no Michael acceptor moiety was almost as active as compound **15**. Since **27** is less toxic it presents an attractive new lead structure derived from nature for further optimization.

Additionally esters of caffeic and ferulic acid under certain conditions may face the problem of limited bioavailability because of the possible formation of a polar phenolate ion which prevents the compound from penetrating the cell membrane. By omitting the hydroxyl groups and thereby increasing the lipophilicity this problem should be avoided.

Since bornyl hydroxycinnamic esters have been shown to inhibit the trypanosomal rhodesain [10] it is tempting to speculate whether they are able to inhibit the corresponding leishmanial proteases. Preliminary experiments using *L. major* promastigote full-lysate in a cysteine-cathepsin fluorescence activity assay [16,34] showed protease inhibitory activity of compound **1** and **15** and, thus, point to this target.
